# Gender representation in Canadian surgical leadership and medical faculties: a cross-sectional study

**DOI:** 10.1186/s12909-024-05641-6

**Published:** 2024-06-17

**Authors:** Lauren Pickel, Nirojini Sivachandran

**Affiliations:** 1 Temerty Faculty of Medicine, University of Toronto, Toronto, Canada; 2 Toronto Retina Institute, Toronto, Canada; 3 Department of Chemistry and Biology, Toronto Metropolitan University, Toronto, ON Canada

**Keywords:** Surgical education, Medical education, Women in medicine, Surgery, Gender, Leadership, Professorship, Department head, Division head

## Abstract

**Background:**

Over the past two and half decades, Canadian medical school students have become majority female, and the medical workforce is therefore increasingly comprised of female physicians. Whether this change, however, has been reflected in the gender balance within medical school faculty positions and leadership has not been well studied in Canada.

**Methods:**

This cross-sectional study examined the genders of full-time faculty members from the most recently available AFMC data, the current heads of departments of medicine and surgery from department websites and confirmed with respective universities.

**Results:**

Overall, women held 40.5% of full-time faculty positions in Canadian faculties of medicine. Female representation decreased with increasing academic rank, from 57.8% of instructors to 50.8% of assistant, 39.2% of associate, and 28.1% of full professors, respectively, with the greatest rate of increase over the past decade among full professors (0.75% per year). The heads of departments of family medicine were majority female (67%), and heads internal medicine at parity (50% female), consistent with numbers of practicing physicians. However, the heads of surgical divisions were majority male (86% overall). Accounting for the gender balance of practicing surgeons, male compared to female surgeons were 2.9 times as likely to be division head (95% CI 1.78–4.85, *p* < 0.0001).

**Conclusions:**

Women remain underrepresented in Canadian faculties of medicine in leadership positions. Leadership in departments of surgery has particularly low female representation, even relative to the proportion of practicing female surgeons within the respective discipline.

## Introduction

Female representation in medicine has greatly risen over the past decades, and in Canada more rapidly than in the US. Whereas gender parity among medical school graduates did not occur in the US until 2021 [1], it was achieved in Canada in 1996, with recent graduating cohorts being nearly 60% female [2, 3]. Nevertheless, around the world gender disparities in medicine remain, with women less likely to hold academic leadership positions [4, 5]. From the gender balance of graduating medical doctors in recent decades, one might predict there to be greater female representation in the leadership of Canadian compared to US faculties of medicine and surgery. However, existing literature has identified several factors which disproportionately influence the career trajectories of women in medicine, including competing responsibilities outside the workplace [6–8], experiences of discrimination [9, 10], and lack of mentorship [11–14]. Whereas the status of women in academic medicine has been systematically reported in the US since 1983 [15], the same is not true in Canada. Existing work has focused on specific specialties such as psychiatry [16], radiology [17], orthopedic surgery [18], and nuclear medicine [19]. In the Canadian context of gender parity having been surpassed in graduating medical students nearly three decades ago, the present study asks whether this has translated to greater representation of women in academic medical leadership and across specialties. We aim to compare the representation of men and women in academic medical leadership positions including full-time faculty, heads of departments of medicine and surgery, and Deans of medicine at Canadian medical schools.

## Methods

The number of faculty of medicine stratified by gender was retrieved from the AFMC Faculty Members Study, available from 2010/11 to 2020/2021, which is derived from annual surveys of Canadian faculties of medicine conducted by the AFMC. To investigate female academic leadership in Canadian medical schools, heads of the largest departments (Family Medicine, Internal Medicine, and Surgery) were compared using publicly available information from department websites at each of Canada’s 14 anglophone faculties of medicine. Given notable differences in female representation within departments of surgery as compared to medicine, the heads of individual surgical divisions were further investigated. Historical information on department heads were not consistently available, therefore this was considered only cross-sectionally. Comparable positions were considered where naming conventions differed between institutions, though we cannot exclude that there may be differences between institutions in the role and process of appointing these positions.

All information on department and division heads was confirmed to be correct and up to date as of April 2023 through direct communication with the respective university. When multiple people held a position, both were counted. Gender (man or woman) was determined according to profiles on the university website, or when unavailable, through their profile on affiliated hospital websites, through pronouns used to describe the faculty member, name, and recent photographs. Deans of medicine were compiled from department websites from all 17 Canadian faculties of medicine. The most recent physician workforce data was obtained from the Canadian Institute for Health Information (CIHI) Scott’s Medical Database to 2022, which is curated using data from medical schools, jurisdictional registrars, the Royal College of Physicians and Surgeons of Canada, and the College of Family Physicians of Canada. Data sources captured male and female as gender, and we have therefore retained this terminology. Data were compiled in Excel, linear regression and odds ratio calculations were performed in R, and plots were created in R and GraphPad Prism.

## Results

**Fig. 1:**
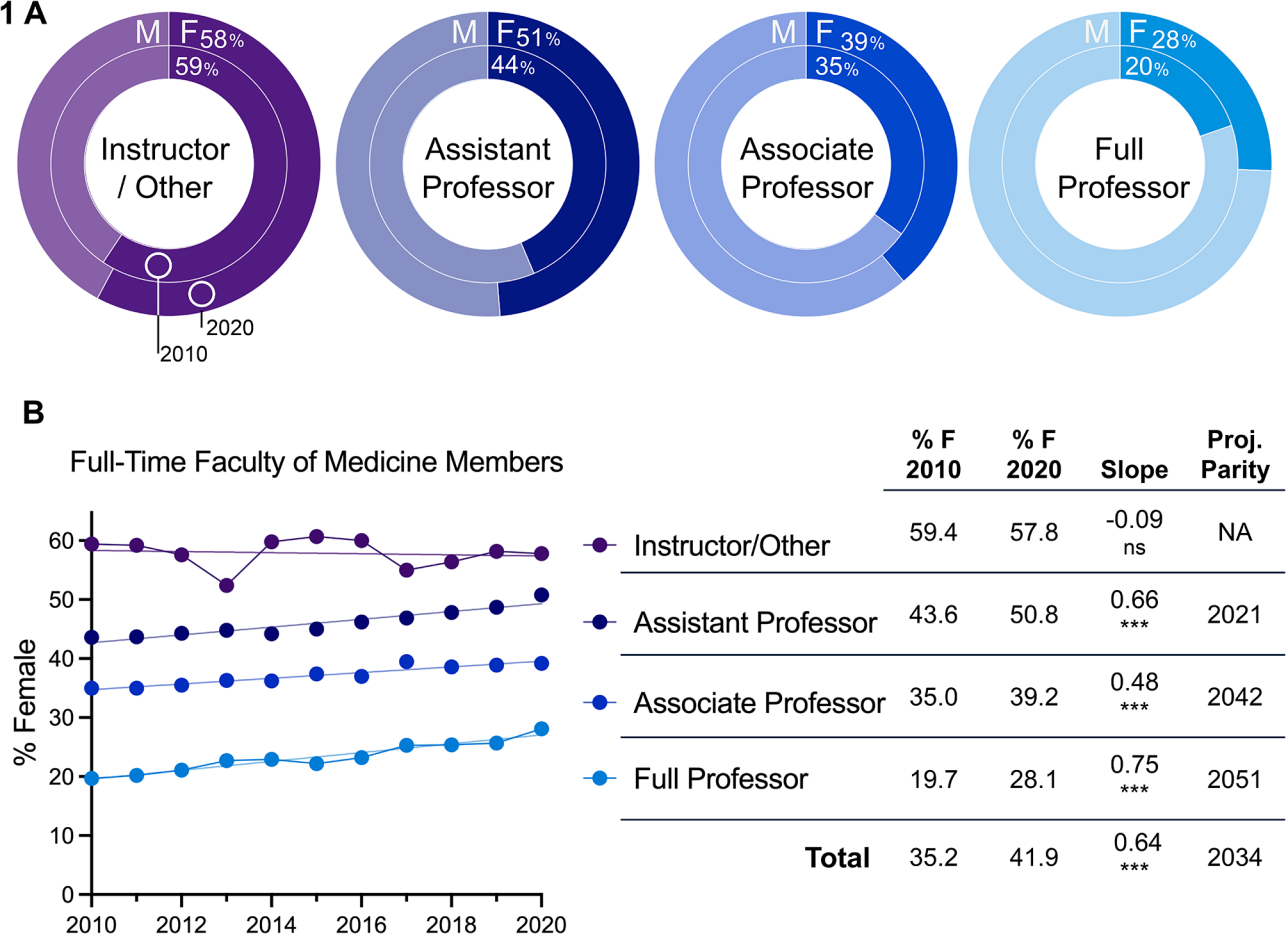
(**A**). Percent female full-time faculty of medicine members by position in 2020/2021 (outer circle) vs. 2010/11 (inner circle). (**B**). Trends in female representation in faculty positions. Year of parity projected where slope of regression differed from zero * *p* < 0.05, ** *p* < 0.01, *** *p* < 0.001. Data from the AFMC

From a total of nearly fifteen thousand full-time faculty of medicine members across Canada, women consistently made up the majority of those in positions of Instructor or Other (57.8%, Fig. 1A). A smaller proportion of women were in Assistant Professor positions (50.8%), though this number significantly grew over the past decade (rate of 0.66% per year), reaching parity in the most recent available data from 2020 to 2021 (Fig. 1B). The proportion of women were in Associate Professor positions was (39.2%), though again growth was significant (0.48% yearly). The lowest female representation was in positions of Full Professorship (28.1%), but this was also the position with the fastest growth (0.75% per year). At the present rate, parity is projected in associate professorships in 2042 and full professorships in 2051.

**Fig. 2:**
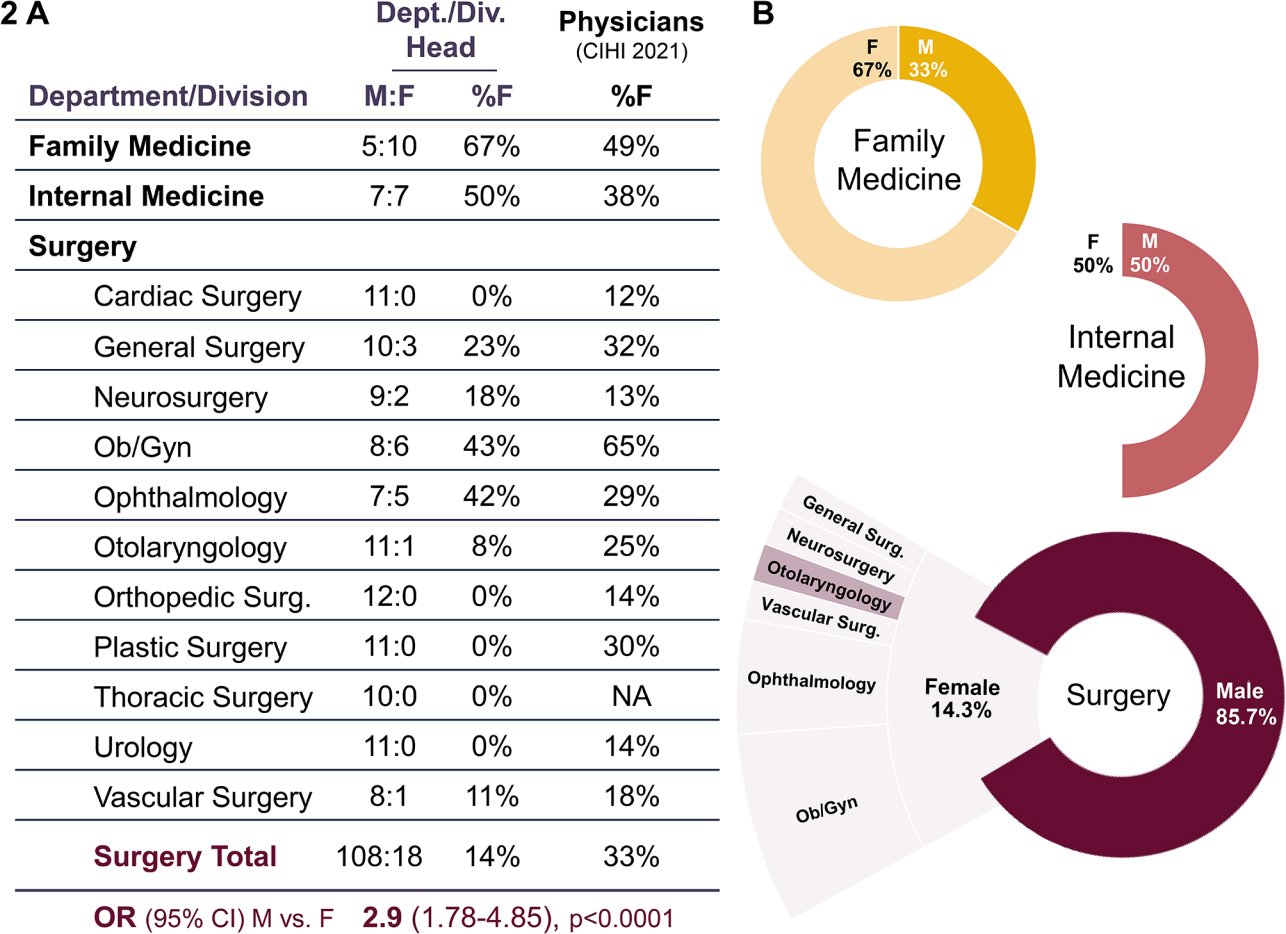
(**A**). Gender distribution of heads of departments and surgical divisions at Canadian faculties of medicine in 2023, as compared to the most recent data on practicing physicians (CIHI, 2021). Heads were confirmed with the respective department. Odds shown for the likelihood of male vs. female practicing surgeons being head of a surgical division, relative to the gender balance of practicing surgeons. (**B**). Illustration of gender balance of heads by department and surgical division

Considering that female representation decreases with increasing academic rank, we then sought to investigate female representation in senior leadership as heads of the departments of family medicine, internal medicine, and divisions of surgery (Fig. 2A). As of 2023, heads of Canadian departments of family medicine were majority female (67%), while internal medicine was evenly split between genders (50% female). Within surgery, division heads were majority male in all specialties (Fig. 2B). The surgical specialties with the greatest proportion of female division heads were Obstetrics and Gynecology (43%), Ophthalmology (42%), General Surgery (23%), and Neurosurgery (18%). On the other hand, there were no female division heads of Cardiac Surgery, Orthopedic Surgery, Plastic Surgery, Thoracic Surgery, or Urology. The proportion of female heads of departments of family and internal medicine are higher compared to the most recent data on practicing physicians [20], though the odds of practicing physicians being department heads in family medicine (M vs. F 0.48, 0.16–1.41) or internal medicine (0.60, 0.21–1.73) did not significantly vary by gender. On the other hand, the representation of women in leadership within divisions of surgery is half the proportion of female practicing surgeons. Accounting for the gender balance of practicing surgeons, the odds of a male surgeon being a division head were 2.9 times that of a female surgeon (95% CI 1.78–4.85, *p* < 0.0001).

**Fig. 3:**
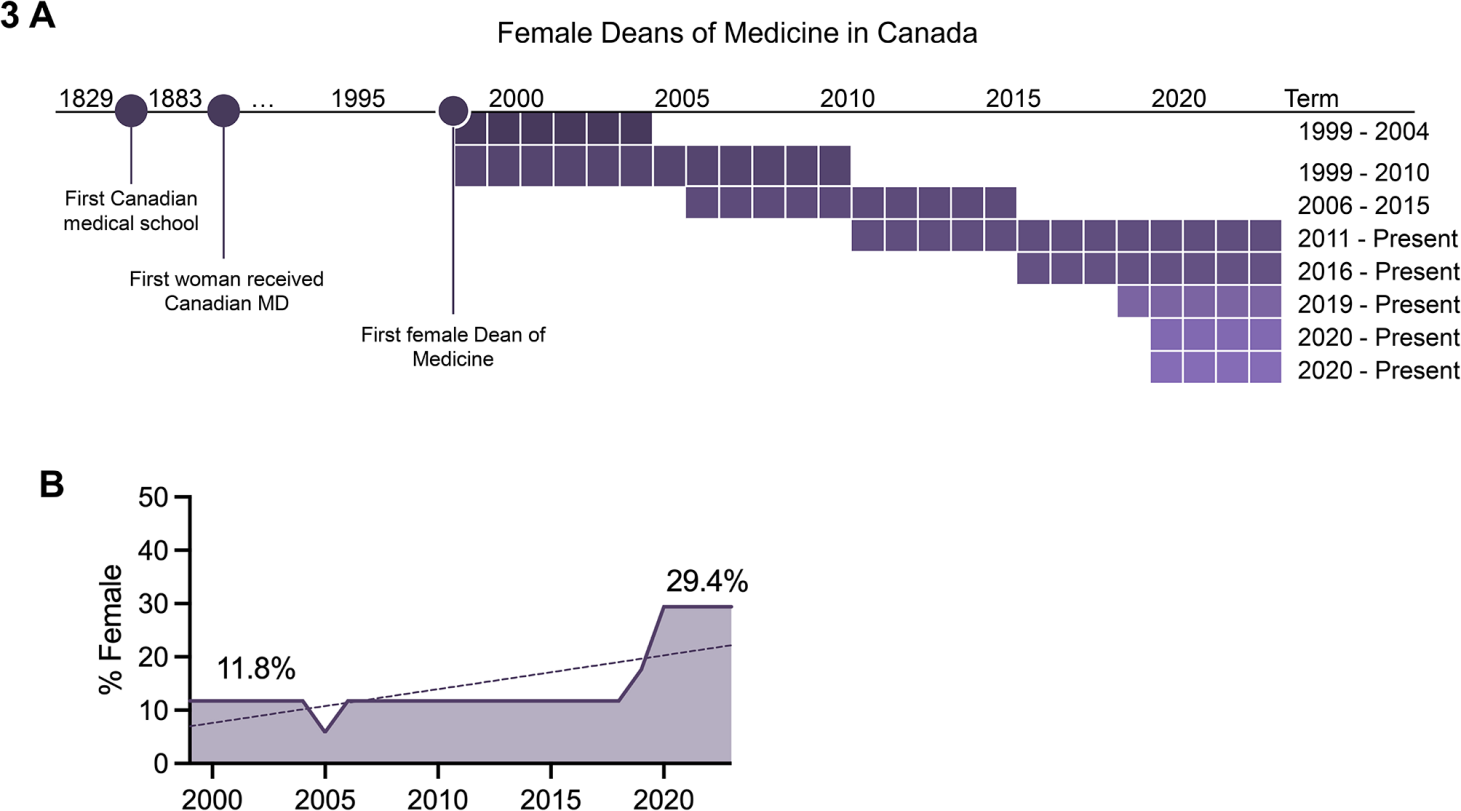
(**A**). Timeline of all women acting as Dean of Medicine in Canada. Rows represent individual Deans. (**B**). Proportion of Deans of Medicine who were women by year

Lastly, all female Deans of Canadian faculties of medicine were compiled (Fig. 3). Today, five of Canada’s seventeen medical schools (29.4%) currently have female Deans, and there have been a historical total of eight female Deans of Medicine. The first began their terms in 1996, more than a century after the first woman graduated from a Canadian medical school [21].

## Discussion

The results of the present study confirm that the representation of women in academic medicine and positions of leadership in Canada remains low compared to the large numbers of female medical students graduating in recent decades. Among full-time faculty positions, contract workers are more often female. Among professors, as academic rank increases from assistant to associate and to full professor, the proportion of women steadily decreases, from nearly half of assistant professors to a quarter of full professors. Notably, Canadian medical school graduates have been at least 50% female since 1996, while in the United States graduating classes did not reach parity until 2021 [1]. Despite this trend, the proportion of female faculty at Canadian medical schools is strikingly similar to the United States, where in the most recent comparable data (2019): 41% (vs. 41% Canadian) of full-time medical school faculty, 58% (vs. 59%) of instructors, 46% (vs. 49%) of assistant professors, 37% (vs. 39%) of associate professors, and 25% (vs. 26%) of full professors were female [15]. The advancement of women in Canadian medical careers has not kept pace with the increasing female representation among trainees.

At the senior leadership level on the other hand, department chairs are more often female in Canada compared to the United States in Family Medicine (67% vs. 30.4%), Internal Medicine (50% vs. 17.7%) as well as Surgery (14% vs. 6.3%) [15]. There is also promise that the pattern of fewer women among higher-ranked faculty positions is changing, as the rate of growth in female representation over the past decade among Canadian faculty was greatest for full professors. Based on the present trends, gender parity is predicted among full professors in 2051, among associate professors in 2042. Gender parity among assistant professors was reached in 2021. The number of female Deans has also dramatically risen in the past decade, from the first and only female Dean acting until 2015, to four current female Deans, of whom three were appointed in the past 5 years.

Along with vertical patterns of underrepresentation in leadership, horizontal disparities in gender representation have been convincingly demonstrated between medical school departments. It was recently shown that the odds of promotion to full professorship in Canadian departments of general surgery were higher for men (OR 2.79, CI 1.13–6.92) even after controlling for number of publications, fellowships, graduate education, and years in practice [22]. Among surgical subspecialties, men occupy 85% of full professorship and 76% of all faculty positions in Canada [23]. We find a similar pattern among heads of departments, which were between 54% and 100% male, with male surgeons being threefold more likely to hold these positions after accounting for the gender balance of practicing surgeons. Divisions of Obstetrics and Gynecology (43% female department heads) and Ophthalmology (42%) were closest to parity, whereas divisions of Cardiac Surgery, Orthopedic Surgery, Plastic Surgery, Thoracic Surgery, and Urology had no female department heads.

Several reasons for disparities in the careers of men and women in medicine have been addressed in previous literature which may in part account for these results. Women in medicine may face disproportionate challenges that contribute to these ongoing gender disparities within medical careers, which include increased time devoted to responsibilities outside the workplace. In a study of active academic physicians holding NIH K08 or K23 grants, women reported 8.5 h more time spent on domestic tasks each week, independent of work hours [6]. When both partners worked, women were more likely to take time off during disruptions to child-care arrangements [6]. Similarly, female surgeons are less likely to have spouses who take on a primary childcare role [7], and more likely to experience a “work-home life conflict” or to report that having children slowed their career advancement [8]. Implicit bias also plays a major role. Interestingly, when an implicit association test revealed the unconscious bias of “male + career” and “female + family” among a majority of plastic surgeons, both male and female, many stated they were unsurprised [24]. Competing responsibilities and implicit biases may contribute to the reduced representation of women in academic positions and leadership within surgical divisions.

Experiences of gender discrimination also continue to impact women at all stages of training, perhaps particularly within surgical fields, though this may not always be easily uncovered. Female academic surgeons clearly expressed personal experiences of discrimination in a focus group, but a pattern of unwillingness to identify as having experienced discrimination or inequity emerged in 1:1 interviews [9]. This distancing is likely an unconscious strategy to mitigate professional harm, though it also demonstrates the ongoing influence of covert discrimination, which affects women throughout training and into practice [10]. When the friction experienced by female trainees and surgeons is disproportionate, the relative number of women reaching positions of leadership in these fields will likely remain small. We also know that female medical students are significantly less likely to enter surgical residencies [3]. These early career decisions are influenced by a variety of factors, with the limitation of female representation in faculty and leadership positions within certain fields, as demonstrated in the present study, likely playing a role.

Mentorship plays a crucial role at every stage of the medical career path, helping women to navigate the unique challenges they face toward achieving their professional goals. Mentorship has been shown to improve career progression and satisfaction for women in academic medicine, yet female physicians are less likely to report having a mentor [11–13]. The absence of women in leadership positions is likely to be detrimental to trainees. Given the low female representation in departments of surgery, it is not surprising that female surgeons are especially unlikely to be able to have a gender-concordant mentor, though it is promising to note this is changing with each entering generation of surgeons being more likely to have a female mentor identified [14]. The slow but steady increase in the representation of women in leadership roles within academic medicine and surgery creates a positive cycle, as female trainees are increasingly able to access mentorship and role models.

### Limitations

The present study has limitations. First, the gender balance of department heads was considered only cross-sectionally, as historical information was not consistently available. Second, the structure of faculties of medicine differed slightly between universities. Though comparable positions were considered wherever possible, there may be differences between institutions in the role and process of appointing these positions. Additionally, data from external sources including the CIHI and AFMC were limited to the most recent versions of the respective surveys. Third, binary gender categories were used, and we were therefore unable to assess differences in academic rank for physicians not conforming to these categories. Finally, the present study is descriptive of the current state of gender balance in Canadian faculties of medicine, but the reason for observed differences cannot be inferred. Future work including surveys or focus groups with Canadian medical trainees and practicing physicians and surgeons is needed to better understand the underlying factors influencing the career decisions of women in medicine.

## Conclusions

This cross-sectional study found that women remain underrepresented in higher ranked academic positions of associate and full professor, and in senior leadership positions within surgical disciplines. While female representation in academic leadership is increasing, the rate of increase has not kept pace with the gender composition of medical trainees. Relative to the large numbers of women entering medical school over the past three decades, with gender parity among Canadian medical graduates having occurred in 1996 and having been consistently surpassed since 2003 [3], the slow increase in proportions reaching leadership positions is notable and speaks to ongoing barriers to career progression. Moreover, it highlights the need for concerted efforts to monitor women’s representation in academic medicine and leadership.

Canadian medical school classes have uniquely high representation of women, but this does not necessarily translate to the equitable advancement of women to positions of leadership within medicine and across disciplines. Leadership positions within academic medicine and surgery are reached through decades of dedicated work, with each stage of training presenting challenges that may uniquely impact women, as well as other historically underrepresented groups. The medical community has a responsibility to identify and address barriers that disproportionately hinder the career progression of certain groups in order to create a medical workforce that is diverse at all levels and across specialties, and therefore equipped to provide equitable care to a diverse patient population.

## Data Availability

The datasets generated by the present study are available from the corresponding author (L.P.) on reasonable request.
